# Managing anticoagulation in a patient with Calciphylaxis and mechanical valve: a therapeutic dilemma

**DOI:** 10.1093/omcr/omaf215

**Published:** 2025-10-29

**Authors:** Qutaiba Qafisheh, Abdalhakim Shubietah, Mohanad Qwaider, Muath A Baniowda, Abdalrahman Assaassa, Roaa Aljunaidi, Abubakar Nazir

**Affiliations:** Department of Medicine, University of Toledo, 3000 Arlington Avenue, Toledo, OH 43614-2595, USA; Department of Medicine, Advocate Illinois Masonic Medical Center, 836 West Wellington Avenue, Chicago, IL 60657, USA; Department of Medicine, University of Toledo, 3000 Arlington Avenue, Toledo, OH 43614-2595, USA; Department of Medicine, University of Missouri–Kansas City School of Medicine, 2411 Holmes Street, Kansas City, MO 64108, USA; Department of Cardiology, Thomas Jefferson University Hospital, 111 South 11th Street, Philadelphia, PA 19107, USA; Faculty of Medicine, Palestine Polytechnic University, Wadi al-Harieh, P.O. Box 198, Hebron, Palestine; Department of Research, Oli Health Magazine Organization, Kigali, Rwanda; Department of Medicine, The Jewish Hospital-Mercy Health, 4777 East Galbraith Road, Cincinnati, OH 45236, USA

**Keywords:** Calciphylaxis, end-stage renal disease, warfarin, sodium thiosulfate, anticoagulation, vascular calcification

## Abstract

**Background:**

Calciphylaxis is a rare, life-threatening vasculopathic syndrome most commonly seen in end-stage renal disease (ESRD) and associated with high mortality. Vitamin K antagonists complicate management in patients with mechanical valves by potentially exacerbating vascular calcification while remaining essential for thromboprophylaxis.

**Case Presentation:**

A 50-year-old woman with ESRD on hemodialysis and a mechanical mitral valve on warfarin presented with severe abdominal pain and rapidly progressive necrotic ulcers. Despite broad-spectrum antibiotics and sodium thiosulfate, lesions worsened. Biopsies were nondiagnostic; however, the clinical phenotype was consistent with calciphylaxis. A multidisciplinary team transitioned anticoagulation from warfarin to subcutaneous unfractionated heparin (UFH) (333 U/kg bolus, then 250 U/kg every 12 h). Anticoagulation was monitored with serial PTTs. Over two months, wounds stabilized with interval debridements, granulation improved, and no valve-related thromboembolic or bleeding complications occurred.

**Conclusion:**

In a mechanical-valve patient with ESRD and suspected calciphylaxis, sustained, full-dose subcutaneous UFH served as a pragmatic alternative to warfarin, allowing wound stabilization without short-term valve complications. Early recognition, multidisciplinary coordination, and individualized anticoagulation strategies are critical to balance thrombotic and calciphylaxis risks.

## Introduction

Calciphylaxis is a rare but life-threatening condition characterized by vascular calcification and thrombosis, predominantly affecting patients with end-stage renal disease (ESRD) on dialysis [1]. It leads to painful skin ulcers and necrosis, often resulting in poor outcomes, including sepsis and death. Warfarin, commonly used in patients with mechanical heart valves, presents a therapeutic dilemma. While it is essential for anticoagulation after valve replacement, it is also a known risk factor for vascular calcifications and calciphylaxis. Managing anticoagulation in such patients requires a delicate balance between preventing thromboembolic events and reducing the risk of calciphylaxis [2].

This report discusses a 50-year-old female with multiple comorbidities, including ESRD and a recent mitral valve replacement, who developed extensive necrotic skin lesions suspected to be calciphylaxis. To our knowledge, prior mechanical-valve calciphylaxis cases have used IV UFH, LMWH, or off-label DOACs; we report sustained, full-dose subcutaneous UFH as the maintenance anticoagulant with two-month follow-up without valve complications.

## Case presentation

A 50-year-old female with a history of insulin-dependent type 2 diabetes, primary hypertension, hyperlipidemia, chronic heart failure with reduced ejection fraction (30–35%), mitral valve replacement and tricuspid valve repair in 2024, patent foramen ovale closure, non-obstructive coronary artery disease, ESRD on hemodialysis, hypothyroidism, obstructive sleep apnea, and a prior ischemic stroke presented to the hospital with severe excoriating abdominal wall pain and ulcerations. Initial laboratory findings are summarized in Table 1.

**Table 1:** Laboratory test results.

Lab Test	Result	Normal Range
White Blood Cell count	9.5 x 10^3/uL	4.5–11.0 × 10^3^/uL
Hemoglobin	8.9 g/dL	12.0–16.0 g/dL
Alanine Aminotransferase	5 IU/L	7–56 IU/L
Aspartate Aminotransferase	8 IU/L	10–40 IU/L
Phosphorus	3.9 mg/dL	2.5–4.5 mg/dL
C-reactive protein	14.4 mg/L	< 10 mg/L
Erythrocyte Sedimentation Rate	51 mm/h	< 20 mm/h
Cytoplasmic Antineutrophil Cytoplasmic Antibodies	Negative	Negative
Perinuclear Antineutrophil Cytoplasmic Antibodies	Negative	Negative
Antinuclear Antibody	Negative	Negative
Anti-double-stranded DNA Antibodies	Negative	Negative

Approximately two weeks before her admission, the patient noticed a small boil on her abdomen. Over the following weeks, this lesion expanded to involve a large portion of the abdominal wall, accompanied by blistering that progressed to necrotic, eschar-like lesions. She did not report significant drainage but experienced fever and chills. A course of outpatient doxycycline failed to improve her symptoms, and she sought further care due to worsening pain.

Upon admission, she was started on vancomycin, cefepime, and oral metronidazole to address suspected panniculitis. A shave biopsy of the abscess revealed a small focus of vasculitis, while a deep skin biopsy of the abdominal wall showed no evidence of dermal organisms on PAS or Gram staining. Despite two weeks of broad-spectrum antibiotic therapy, new lesions developed on her left lateral hip area, and her abdominal lesions showed no improvement. The skin lesions, characterized by intensely painful, necrotic ulcers that progressed to indurated nodules and black eschars due to tissue necrosis, raised suspicion for calciphylaxis, prompting initiation of sodium thiosulfate. However, a deep skin punch biopsy revealed epidermal and subcutaneous necrosis with increased vascular proliferation but no histological evidence of calciphylaxis.

The patient’s case presented a challenging dilemma. Warfarin had been maintained with a target INR of 2.5–3.5 due to her mechanical mitral valve, but given the increased risk of calciphylaxis and its association with poorer outcomes, a change in management was warranted. A multidisciplinary team—comprising specialists in hematology, cardiology, cardiothoracic surgery, nephrology, internal medicine, and infectious disease—collaborated on her care. While the skin biopsy revealed no definitive evidence of calciphylaxis, the team agreed that her clinical presentation was consistent with calciphylaxis. The consensus was to switch her anticoagulation from warfarin to unfractionated heparin (UFH) during her hospitalization. She was later transitioned to subcutaneous UFH, initiated with a bolus of 333 units/kg, followed by 250 units/kg administered every 12 h.

The patient’s anticoagulation was carefully monitored with weekly PTT levels, and she was followed up in the office after two months. During this period, her PTT remained within the therapeutic range. At follow-up, the patient’s lesions showed stabilization/regression in the two wounds she has, and her overall condition improved.

**Figure 1:**
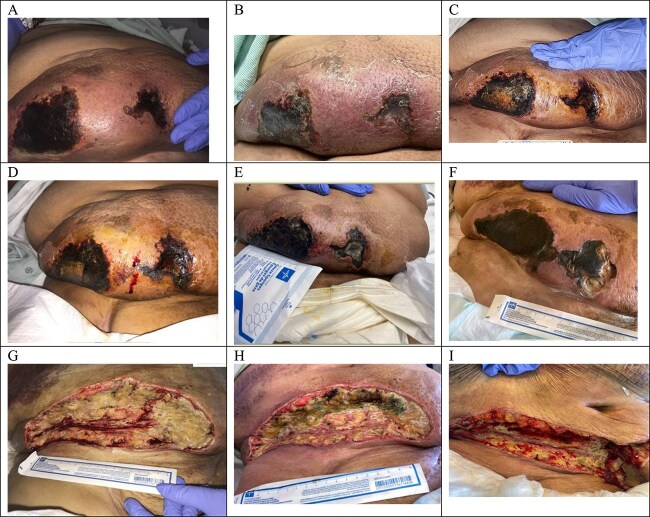
Progression of abdominal wound from necrotic ulceration on warfarin to stabilization after transition to subcutaneous unfractionated heparin (UFH). The wound progression began with image a, showing extensive necrotic ulcers while the patient was on warfarin. Two weeks later (image B), there was worsening ulceration and increased tissue scaling, prompting a switch to subcutaneous heparin. One month later (image C), the wound stabilized without further ulceration or deepening. By two months (image D), the wound remained stable with minimal superficial bleeding. The patient was later admitted with sepsis (image E), likely from wound infection, requiring surgical debridement. Image F shows the wound pre-debridement, and image G shows the immediate post-debridement bed. Image H (10 days later) revealed reinfection, necessitating a second debridement (image I).

**Figure 2:**
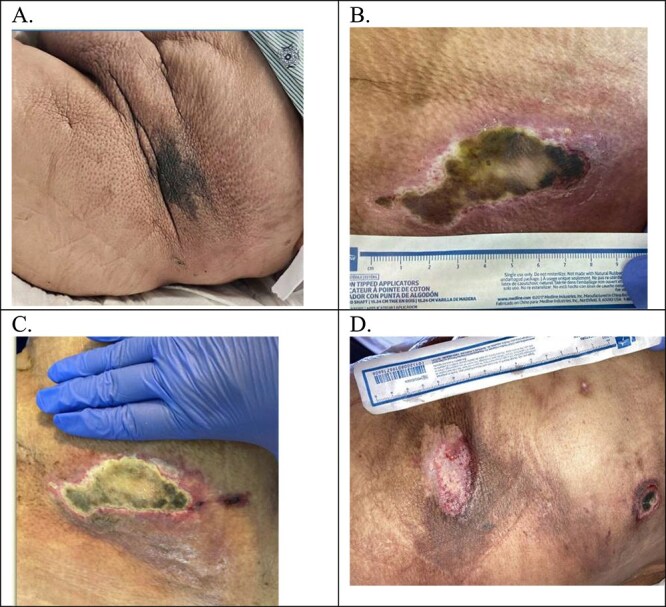
Progression of left flank wound over the course of subcutaneous unfractionated heparin (UFH) therapy. The left flank lesion initially appeared as a small, dark, indurated patch (image a). By the time of transition from warfarin to subcutaneous heparin (image B), the lesion had progressed to an ulcer with central necrosis and surrounding erythema, indicating early tissue breakdown. One month after switching to subcutaneous heparin (image C), the wound showed stabilization with well-demarcated edges and no further deepening. At two months (image D), there was marked improvement, with healthy granulation tissue replacing necrotic areas and an overall reduction in wound size, suggesting a favorable healing trajectory under the adjusted anticoagulation regimen.

Approximately 2.5 months following her initial presentation, the patient was readmitted with a wound infection that rapidly progressed to sepsis. Despite intensive medical management, her clinical condition deteriorated, and she ultimately succumbed to sepsis-related complications. While the patient’s outcome was fatal due to infectious sequelae, the use of subcutaneous UFH effectively prevented short-term valve-related thromboembolic events and contributed to stabilization of the calciphylaxis-related wounds. Figure 1(A–I) Illustrates the abdominal calciphylaxis wound trajectory: Extensive necrotic ulceration on warfarin (A), interval worsening prompting transition to subcutaneous unfractionated heparin (UFH) (B), subsequent stabilization without further deepening (C and D), and episodes of infected breakdown requiring surgical debridement with an improved post-debridement bed (E–I). Figure 2(A–D) Shows a concordant course in the left-flank lesion—From early indurated plaque and ulceration on warfarin (A and B) to demarcation, granulation, and overall size reduction over two months after switching to subcutaneous UFH (C and D).

## Discussion

Calciphylaxis is a rare but severe condition characterized by vascular calcification and thrombosis, leading to the blockage of small blood vessels in the subcutaneous fat and dermal layers, resulting in painful ischemic skin ulcers. This condition has a grim prognosis, with a survival rate typically less than one year in most cases [3–5]. While ESRD is the most significant risk factor, calciphylaxis can also occur in non-uremic patients. Other contributing factors include obesity, diabetes, and anticoagulant use, particularly warfarin, which is considered one of the strongest predictors of mortality in these patients [6].

Painful skin lesions are observed in 90–100% of calciphylaxis cases, often accompanied by gangrene (10–25%) and a high risk of wound infection or sepsis (40–50%). Sepsis remains the leading cause of death in these patients [4, 7]. While a deep skin biopsy is the gold standard for diagnosis, it may be inconclusive in 20–40% of cases. In such instances, clinicians must rely on clinical presentation, imaging, and risk factor assessment to confirm the diagnosis when biopsy results are non-diagnostic or impractical [8].

Our patient had been on dialysis for two years, consistent with literature reporting an average onset of calciphylaxis around 2.5 years after initiating dialysis. Six months prior to the onset of calciphylaxis, the patient was started on warfarin following a mechanical mitral valve replacement, aligning with known risk factors for calciphylaxis.

Management involves comprehensive wound care, pain control, risk factor modification, and pharmacological interventions. Wound care often includes surgical debridement; however, in this case, surgical options were limited due to extensive involvement of the hip, abdomen, and sacral areas [9]. Pharmacological treatments frequently employed include sodium thiosulfate and vitamin K. Sodium thiosulfate shows promise in treating calciphylaxis by binding calcium ions to form soluble complexes, thereby preventing calcium phosphate precipitation and alleviating pain. Despite its potential, the effectiveness of sodium thiosulfate is variable and often requires prolonged off-label use [10]. Sodium thiosulfate may also enhance vascular endothelial function, promote vasodilation, inhibit vascular smooth muscle cell proliferation, and support hepatic production of proteins like albumin and fetuin-A [11].

Anticoagulation therapy presents significant challenges in managing calciphylaxis. Warfarin, essential for patients with mechanical heart valves, is linked to increased mortality in calciphylaxis [12]. Direct oral anticoagulants (DOACs) are not recommended in mechanical valve patients due to elevated thromboembolic and bleeding risks [13]. The PROACT Xa trial highlighted this concern by reporting higher thromboembolic events in participants with an On-X mechanical aortic valve who received apixaban compared to those receiving warfarin [14].

Subcutaneous UFH provides an alternative, particularly in ESRD patients, as it is not renally excreted. Evidence suggests it is as effective and safe as LMWH for venous thromboembolism treatment, with no significant differences in recurrent thromboembolic events or major bleeding over a 3-month follow-up [15, 16]. However, maintaining therapeutic levels can be challenging, as illustrated by Berry et al., where exceedingly high doses were required, making UFH use impractical. In their case, warfarin was continued despite the risks because of the high thrombosis risk associated with two mechanical heart valves [17].

One case reported the successful use of intravenous UFH in an ESRD patient with calciphylaxis. The heparin dose was titrated to an aPTT of 1.5–2 times baseline, leading to complete healing of a penile ulcer over eight weeks. Warfarin was cautiously reintroduced without recurrence of calciphylaxis over six months [18]. Another report by Nyembo et al. documented rapid development of calciphylaxis in a patient with ESRD following dual mechanical valve replacement and warfarin initiation. Due to UFH’s limitations, an interdisciplinary team transitioned the patient to apixaban, despite its off-label use for mechanical valves [19].

The choice of prosthetic valves in ESRD patients presents unique challenges. Bioprosthetic valves are prone to rapid degeneration due to mineral metabolism abnormalities associated with ESRD. Conversely, mechanical valves require lifelong anticoagulation, increasing the risk of bleeding complications and calciphylaxis [20]. The potential role of bioprosthetic valves in avoiding metastatic calcification associated with warfarin use in mechanical valve replacements warrants further consideration.

Although the patient ultimately died from sepsis-related complications, this outcome does not imply that the use of subcutaneous UFH was ineffective. On the contrary, during the short-term period following the anticoagulation switch, the patient demonstrated notable clinical improvement, including stabilization of skin lesions, absence of new deep wound progression, and no valve-related thromboembolic or bleeding events. These observations suggest that UFH was a viable short-term alternative to warfarin in managing anticoagulation for a mechanical valve in the setting of calciphylaxis. However, the lack of long-term follow-up data limits the ability to assess the sustained efficacy and safety of this approach. Future studies or case series with extended observation are warranted to better understand the long-term outcomes of subcutaneous UFH in similar patient populations.

## Conclusion

Calciphylaxis is a rare but severe condition in ESRD patients with mechanical heart valves on warfarin, posing significant anticoagulation challenges. Limited evidence exists on alternative anticoagulants or optimal prosthetic valves, necessitating careful valve selection and individualized anticoagulation to mitigate risks. Early recognition and multidisciplinary coordination are critical to preventing complications such as sepsis and death. To our knowledge, this is the first report describing sustained, full-dose subcutaneous unfractionated heparin as maintenance anticoagulation in an ESRD patient with a mechanical valve who developed calciphylaxis on warfarin. A key limitation is the lack of long-term follow-up: although the wound stabilized without further progression after transition to subcutaneous UFH, the patient ultimately died from sepsis secondary to wound infection, precluding assessment of durability and late valve-related outcomes.

## References

[1] Nigwekar SU, Thadhani R, Brandenburg VM. Calciphylaxis. Ingelfinger JR, editor. N Engl J Med 2018;378:1704–14. 10.1056/NEJMra1505292 29719190

[2] Noh H, Yu MR, Kim HJ. et al. Beta 2-adrenergic receptor agonists are novel regulators of macrophage activation in diabetic renal and cardiovascular complications. Kidney Int 2017;92:101–13. 10.1016/j.kint.2017.02.013 28396116 PMC5483383

[3] McCarthy JT, El-Azhary RA, Patzelt MT. et al. Survival, risk factors, and effect of treatment in 101 patients with Calciphylaxis. Mayo Clin Proc 2016;91:1384–94. 10.1016/j.mayocp.2016.06.025 27712637

[4] Nigwekar SU, Zhao S, Wenger J. et al. A nationally representative study of calcific uremic Arteriolopathy risk factors. J Am Soc Nephrol 2016;27:3421–9. 10.1681/ASN.2015091065 27080977 PMC5084892

[5] Fine A, Zacharias J. Calciphylaxis is usually non-ulcerating: risk factors, outcome and therapy. Kidney Int 2002;61:2210–7. 10.1046/j.1523-1755.2002.00375.x 12028462

[6] Nigwekar SU, Wolf M, Sterns RH. et al. Calciphylaxis from nonuremic causes: a systematic review. Clin J Am Soc Nephrol 2008;3:1139–43. 10.2215/CJN.00530108 18417747 PMC2440281

[7] Weenig RH, Sewell LD, Davis MDP. et al. Calciphylaxis: natural history, risk factor analysis, and outcome. J Am Acad Dermatol 2007;56:569–79. 10.1016/j.jaad.2006.08.065 17141359

[8] Patel MR, Mahaffey KW, Garg J. et al. Rivaroxaban versus warfarin in Nonvalvular atrial fibrillation. N Engl J Med 2011;365:883–91. 10.1056/NEJMoa1009638 21830957

[9] Roy S, Reddy SN, Garcha AS. et al. Successful treatment of Calciphylaxis in a young female with end-stage renal disease on peritoneal dialysis with Parathyroidectomy, intensification of dialysis, and sodium Thiosulphate—a case report and literature review. J Investig Med High Impact Case Rep 2021;9:1–7. 10.1177/23247096211060580 PMC8637396 34845938

[10] Yang X, Liu Y, Xie X. et al. Use of the optimized sodium thiosulfate regimen for the treatment of calciphylaxis in Chinese patients. Ren Fail 2022;44:914–22. 10.1080/0886022X.2022.2081179 35634730 PMC9154757

[11] Hayden MR, Goldsmith DJA. Sodium thiosulfate: new Hope for THE treatment of Calciphylaxis. Semin Dial 2010;23:258–62. 10.1111/j.1525-139X.2010.00738.x 20636917

[12] Aursulesei V, Costache II. Anticoagulation in chronic kidney disease: from guidelines to clinical practice. Clin Cardiol 2019;42:774–82. 10.1002/clc.23196 31102275 PMC6671778

[13] Vahanian A, Beyersdorf F, Praz F. et al. 2021 ESC/EACTS guidelines for the management of valvular heart disease. Eur Heart J 2022;43:561–632. 10.1093/eurheartj/ehab395 34453165

[14] Wang TY, Svensson LG, Wen J. et al. Apixaban or warfarin in patients with an on-X mechanical aortic valve. NEJM Evid 2023;2. Available from: 10.1056/EVIDoa2300067 38320162

[15] Robertson L, Jones LE. Fixed dose subcutaneous low molecular weight heparins versus adjusted dose unfractionated heparin for the initial treatment of venous thromboembolism. Cochrane vascular group, editor. Cochrane Database Syst Rev 2017;2:CD001100. 28182249 10.1002/14651858.CD001100.pub4 PMC6464611

[16] Robertson L, Strachan J. Subcutaneous unfractionated heparin for the initial treatment of venous thromboembolism. Cochrane vascular group, editor. Cochrane Database Syst Rev 2017;2:CD006771. Available from: 10.1002/14651858.CD006771.pub3;2:CD006771 28195640 PMC6464347

[17] Berry A, Degheim G, Saba S. Arteriolar vs. valvular thrombosis: pick your evil! Thromb J 2018;16:23. 10.1186/s12959-018-0175-3 PMC6114737 30181717

[18] Chan YH, Wong KM, Kwok PCH. et al. Atypical presentation of calciphylaxis in a patient with renal failure: successful treatment with unfractionated heparin. Hong Kong J Nephrol 2001;3:103–6. 10.1016/S1561-5413(09)60067-3

[19] Nyembo PF, Eidman KE, Simegn M. et al. Rapidly progressive and catastrophic Calciphylaxis after mechanical valve replacement. JACC Case Rep 2022;4:1319–23. 10.1016/j.jaccas.2022.07.034 36406917 PMC9666739

[20] Cadavid JC, DiVietro ML, Torres EA. et al. Warfarin-induced pulmonary metastatic calcification and Calciphylaxis in a patient with end-stage renal disease. Chest 2011;139:1503–6. 10.1378/chest.10-1322 21652561

